# A Novel Integrated Perioperative Cardiovascular Risk Score (PERFORM-CV) in Non-Cardiac Surgical Patients

**DOI:** 10.3390/jcdd13040165

**Published:** 2026-04-10

**Authors:** Andreea Boghean, Cristian Gutu, Laura Florentina Rebegea, Dorel Firescu

**Affiliations:** 1 Faculty of Medicine and Pharmacy, Research Centre in the Medical-Pharmaceutical Field, “Dunarea de Jos” University of Galati, 800008 Galati, Romania; laura.rebegea@ugal.ro (L.F.R.); dorelfirescu@yahoo.com (D.F.); 2 Cardiology Department, “Dr. Aristide Serfioti” Emergency Military Hospital, 800150 Galati, Romania; 3 Radiotherapy Department, “Sf. Apostol Andrei” County Emergency Clinical Hospital, 800578 Galati, Romania

**Keywords:** perioperative risk, non-cardiac surgery, echocardiography, MAPSE, in-hospital mortality, PERFORM-CV score

## Abstract

Background: Perioperative cardiovascular risk assessment remains challenging in non-cardiac surgery, particularly in older patients and those with multiple comorbidities. Traditional models rely largely on clinical history and may not fully reflect current cardiovascular functional status. This study aimed to derive and assess the apparent performance of a new composite score, PERFORM-CV, integrating clinical, laboratory, and echocardiographic data. Methods: We conducted a prospective two-center cohort study including 503 non-cardiac surgical patients with cardiovascular comorbidity. The Revised Cardiac Risk Index (Lee/RCRI) and the AUB-HAS2 index were calculated according to their original published definitions as raw point totals ranging from 0 to 6; without additional normalization. The PERFORM-CV score was derived from univariable and multivariable analyses, with continuous predictors dichotomized using ROC-derived thresholds. Results: Emergency admission, chronic heart failure, and elevated serum creatinine remained independently associated with in-hospital mortality. Lower left ventricular ejection fraction, lower mitral annular plane systolic excursion (MAPSE), lower hemoglobin, and atrial fibrillation also contributed to the final composite score. ROC analysis showed good discrimination for PERFORM-CV (AUC 0.852; 95% CI 0.806–0.897; *p* < 0.001), comparable to Lee/RCRI (AUC 0.860; 95% CI 0.818–0.901; *p* < 0.001) and higher than AUB-HAS2 (AUC 0.779; 95% CI 0.731–0.826; *p* < 0.001). Conclusions: PERFORM-CV showed good apparent discrimination in the derivation cohort and may complement established bedside risk tools by incorporating echocardiographic and laboratory data. The ROC-derived thresholds should be interpreted as data-driven derivation cut-offs; resampling-based internal validation and external validation are required before broader clinical use.

## 1. Introduction

Preoperative cardiovascular assessment in patients undergoing non-cardiac surgery remains a cornerstone of perioperative medicine [1]. Despite advances in surgical and anesthetic techniques, major adverse cardiovascular events (MACE) continue to contribute substantially to postoperative morbidity and mortality [2]. Current practice relies on validated risk stratification tools, most notably the Revised Cardiac Risk Index (RCRI/Lee score) and the more recent AUB-HAS2 index. The RCRI is widely used because of its simplicity, and emphasizes clinical history, including ischemic heart disease, heart failure, and renal dysfunction [3,4,5]. Similarly, the AUB-HAS2 index was developed to refine risk prediction by incorporating additional clinical variables, including age and surgical urgency [4,5,6]. These tools remain useful in daily practice because they are rapid and widely applicable; however, they were not designed to capture the full physiological heterogeneity of contemporary surgical populations, especially older patients admitted urgently and those with multiple interacting cardiovascular and metabolic comorbidities.

These scoring systems nevertheless have important limitations [7] and may underestimate risk, particularly in older patients and in those with multiple comorbidities. A key limitation of both the RCRI and AUB-HAS2 is their reliance on static clinical history and baseline laboratory data, often without incorporating objective, real-time physiological information from transthoracic echocardiography. By not accounting for cardiac structure and function, such as left ventricular ejection fraction (LVEF) [8] and mitral annular plane systolic excursion (MAPSE) [9], conventional scores may be less sensitive to subclinical myocardial dysfunction, impaired myocardial reserve, and hemodynamic vulnerability that become clinically relevant during surgical stress.

Clinical history alone may not adequately reflect a patient’s functional capacity under surgical stress [10]. Therefore, an integrated approach combining bedside imaging with clinical and laboratory markers is needed. By incorporating MAPSE, which reflects longitudinal myocardial fiber shortening [9] and may complement LVEF, the proposed PERFORM-CV score aims to improve risk stratification in contemporary non-cardiac surgical populations. In this context, the present study had two objectives: first, to examine the apparent performance of the Lee/RCRI and AUB-HAS2 scores in a real-world cohort of non-cardiac surgical patients with cardiovascular comorbidity; and second, to derive a pragmatic composite score integrating clinical presentation, routine laboratory data, and focused echocardiographic variables to support bedside estimation of in-hospital mortality risk.

## 2. Materials and Methods

### 2.1. Study Design and Population

We conducted a prospective multicenter cohort study with longitudinal perioperative follow-up based on clinical, laboratory, and echocardiographic data. The study included 503 consecutive patients admitted between October 2022 and July 2025 to two surgical centers in Galați, Romania: the Second Surgery Clinic of the “Sf. Apostol Andrei” Emergency Clinical County Hospital and the Department of Surgery of the “Dr. Aristide Serfioti” Military Emergency Hospital. The study was designed to reflect routine perioperative practice in patients with pre-existing cardiovascular disease undergoing non-cardiac surgery and to assess the relationship between baseline cardiovascular burden and in-hospital perioperative outcomes.

### 2.2. Study Population

Eligible participants were adults, including geriatric patients, undergoing non-cardiac surgery with documented cardiovascular disease and/or major cardiometabolic comorbidities. These included arterial hypertension, chronic coronary syndrome, atherosclerosis, chronic heart failure, atrial fibrillation or atrial flutter, diabetes mellitus, and obesity, defined as a body mass index (BMI) >30 kg/m^2^ [11].

The cohort reflected real-world non-cardiac surgical practice and included both elective and emergency admissions. To better characterize the operative case mix, procedures were grouped into clinically relevant surgical categories. However, a more granular classification according to surgical subspecialty, oncologic status, anesthetic technique, perioperative setting, and standardized baseline severity indices was not available in a sufficiently uniform format to support robust subgroup analyses.

### 2.3. Exclusion Criteria

Patients were excluded if they did not undergo the scheduled surgical procedure because the study focused specifically on perioperative risk in operated patients. Patients transferred to another healthcare facility during the perioperative period were also excluded, as complete in-hospital follow-up and outcome ascertainment could not be ensured. In addition, patients with incomplete or unverifiable documentation of pre-existing cardiovascular disease were excluded to minimize misclassification of baseline cardiovascular status.

### 2.4. Outcome Definition and Follow-Up

The primary endpoint was in-hospital mortality, defined as death from any cause occurring during the index hospitalization before discharge. Patients were followed from the date of hospital admission until in-hospital death or discharge alive. Because the timing of death during hospitalization was considered clinically relevant and the duration of hospital stay varied among patients, the primary endpoint was analyzed in a time-to-event framework. Time zero was defined as the date of hospital admission, and time-to-event was calculated as the number of days from admission to in-hospital death. Patients discharged alive were censored on the date of discharge.

### 2.5. Clinical and Laboratory Variables

The following baseline variables were evaluated as potential predictors of in-hospital death: mode of admission (emergency vs. elective admission), chronic heart failure, atrial fibrillation, atherosclerosis, serum creatinine, hemoglobin, blood glucose, left ventricular ejection fraction, and mitral annular plane systolic excursion. These variables were selected on the basis of clinical relevance and their observed association with in-hospital death in the study cohort. In the initial analyses, in-hospital death was significantly associated with emergency presentation, chronic heart failure, atrial fibrillation, atherosclerosis, higher serum creatinine, lower hemoglobin, higher blood glucose, lower ejection fraction, and lower mitral annular plane systolic excursion.

### 2.6. Echocardiographic Assessment 

Preoperative assessment for each participant included laboratory testing and a focused bedside transthoracic echocardiographic evaluation consistent with a point-of-care ultrasound (POCUS) approach. The aim of the preoperative work-up was not only to document known cardiovascular disease but also to identify objective functional markers that may be missed by purely history-based risk scores. This focused echocardiographic strategy was intentionally selected to shorten evaluation time, allow beside assessment, and improve feasibility in routine perioperative practice. The examination focused on readily obtainable parameters with potential prognostic value.

Left ventricular (LV) systolic function was assessed by left ventricular ejection fraction (LVEF), measured by the Simpson biplane method, and by longitudinal systolic function assessed through mitral annular plane systolic excursion (MAPSE), measured by M-mode [12,13,14,15].

LV diastolic function was evaluated using peak early diastolic transmitral inflow velocity (E wave), peak late diastolic transmitral inflow velocity (A wave), the E/A ratio, and E-wave deceleration time (EDT) [12,13,14]. These variables were included because they can be obtained relatively quickly during a focused transthoracic examination and provide a practical characterization of ventricular filling patterns in the perioperative setting. Advanced deformation and filling indices, including global longitudinal strain (GLS), tissue Doppler velocities, and E/e′, were not consistently available in all patients and therefore were not entered into the derivation model.

In addition, perioperative cardiovascular risk stratification was performed for every participant by calculating both the Revised Cardiac Risk Index (RCRI/Lee) and the AUB-HAS2 index using baseline preoperative clinical and laboratory data recorded at admission. The RCRI/Lee score was calculated according to the original Lee definition and includes six predictors: high-risk surgery, history of ischemic heart disease, history of heart failure, history of cerebrovascular disease, preoperative insulin-treated diabetes, and preoperative serum creatinine >2.0 mg/dL [16]. Each predictor was assigned 1 point, yielding a raw total score ranging from 0 to 6; participants were additionally grouped into the conventional RCRI classes (0, 1, 2, and ≥3 points) [16]. The AUB-HAS2 index was calculated according to the original published definition and includes six elements: history of heart disease, symptoms of angina or dyspnea, age ≥75 years, anemia defined as hemoglobin <12 g/dL, vascular surgery, and emergency surgery [6,7]. One point was assigned for each element present, yielding a raw total score ranging from 0 to 6; scores were interpreted as low risk (0–1), intermediate risk (2–3), and high risk (>3) [6,7]. For ROC analyses, both comparator scores were analyzed on their original raw 0–6 scales; no additional normalization or rescaling was applied.

All echocardiographic measurements and qualitative assessments were performed in accordance with current guidelines from the American Society of Echocardiography (ASE) [17,18] and the European Association of Cardiovascular Imaging (EACVI) [17,18]. Normal reference ranges and severity grading for valvular heart disease were applied according to these standards to ensure a uniform approach to identifying systolic and diastolic dysfunction. Although valvular abnormalities were documented in a substantial proportion of patients, no hemodynamically significant valvular lesions were identified on the basis of the available clinical and echocardiographic records. In addition, a standardized quantitative dataset for individual valvular lesions, particularly grading of mitral regurgitation severity, was not available for all participants; therefore, valvular parameters were not incorporated into the final score. All focused echocardiographic examinations were performed by a single operator, which improved procedural consistency across the cohort; however, interobserver re-producibility could not be assessed.

### 2.7. Ethical Considerations 

The study was conducted in accordance with the Declaration of Helsinki and the Code of Ethics of “Dunărea de Jos” University of Galați (UDJG). The protocol was approved by the Institutional Review Boards of both participating hospitals. Written informed consent was obtained from all participants or their legal representatives. Data confidentiality was ensured throughout the study.

### 2.8. Statistical Analysis 

Data were analyzed using IBM SPSS Statistics, version 26 (IBM Corporation, Armonk, NY, USA). Categorical variables are presented as frequencies and percentages and were compared using the chi-square test or Fisher’s exact test, as appropriate. Continuous variables are reported as median (interquartile range, IQR) or mean ± standard deviation, as appropriate, and were compared using the Mann–Whitney U test, Student’s *t*-test, or Kruskal–Wallis H test, as appropriate. Discriminative performance was evaluated using receiver operating characteristic (ROC) analysis and the area under the curve (AUC). Lee/RCRI and AUB-HAS2 were analyzed as raw point totals (range 0–6 for each score), without normalization or rescaling. Predictors of in-hospital mortality were first explored in univariable Cox proportional hazards analyses using preoperative clinical, laboratory, and echocardiographic variables selected on clinical grounds and on the basis of routine perioperative availability. Variables showing statistically significant or clinically meaningful associations were then considered for multivariable Cox proportional hazards modeling to estimate hazard ratios (HRs) with 95% confidence intervals (CIs), while keeping the final model parsimonious relative to the number of events. For the primary endpoint, follow-up time was defined as the number of in-hospital days from admission to death; patients discharged alive were censored on the day of discharge. This time-to-event framework was used because the duration of hospitalization differed across patients. The proportional hazards assumption was not formally assessed; accordingly, the Cox-model estimates should be interpreted as exploratory measures of association in the derivation cohort. Potential redundancy between MAPSE and LVEF was assessed using both Spearman’s rho and Pearson’s correlation coefficient, and collinearity was further explored by calculating the variance inflation factor (VIF). Given the prospective data collection and the exclusion of patients with incomplete or unverifiable documentation, the main analyses were performed on complete available data for the variables included in the model, and no imputation procedures were applied. For derivation of the PERFORM-CV score, continuous variables were dichotomized using ROC-derived thresholds and an additive point-based score was constructed from the retained predictors. Survival curves were generated using the Kaplan–Meier method and compared with the log-rank test. Because the primary endpoint was binary/time-dependent rather than continuous, the main measures of association were hazard ratios and AUCs rather than simple correlation coefficients; however, exploratory Spearman correlation analysis was also used to assess the monotonic association between increasing PERFORM-CV values and vital status. Because the score was derived and tested in the same cohort, the reported discrimination estimates represent apparent derivation-cohort performance rather than formal internal validation. A *p* value < 0.05 was considered statistically significant.

### 2.9. Development of the Prognostic Score

A prognostic score for in-hospital mortality was derived from variables associated with the primary endpoint in the univariable Cox analysis and from dichotomized continuous predictors defined by receiver operating characteristic (ROC) analysis. Variables associated with in-hospital death in the initial analyses were emergency presentation, chronic heart failure, atrial fibrillation, atherosclerosis, increased serum creatinine, lower hemoglobin, higher blood glucose, lower left ventricular ejection fraction, and lower mitral annular systolic excursion.

For continuous predictors, ROC curves were used to identify data-driven thresholds for predicting in-hospital death. The selected cut-offs were serum creatinine ≥1.775 mg/dL, hemoglobin ≤10.25 g/dL, left ventricular ejection fraction ≤54%, and mitral annular plane systolic excursion ≤10.02 mm. Blood glucose did not show a statistically significant threshold and was therefore not retained in the final score.

Score construction followed a prespecified additive rule. Each variable that was statistically significant in the univariable analysis and retained in the final score was assigned 1 point. Variables that also remained statistically significant in the multivariable Cox analysis received an additional 1 point. Accordingly, the final score included seven predictors: emergency presentation (2 points), chronic heart failure (2 points), atrial fibrillation (1 point), serum creatinine ≥1.775 mg/dL (2 points), hemoglobin ≤10.25 g/dL (1 point), left ventricular ejection fraction ≤54% (1 point), and mitral annular plane systolic excursion ≤10.02 mm (1 point). The total score ranged from 0 to 10 points. These ROC-derived component thresholds were used for score derivation and should be interpreted as data-driven derivation cut-offs rather than definitive clinical decision thresholds. For bedside interpretation, the score was also grouped into pragmatic rounded risk categories: low risk (0–2 points), intermediate risk (3–5 points), and high risk (≥6 points).

Although atherosclerosis was associated with the endpoint in univariable analysis, it was not retained in the final score in order to preserve score parsimony and prioritize variables with stronger and more clinically actionable prognostic value.

### 2.10. Assessment of Score Performance

The score was evaluated in the derivation cohort both as an ordinal variable and as a categorical variable. As an ordinal variable, score values were compared between survivors and non-survivors using the Mann–Whitney U test, and discrimination for in-hospital death was assessed by ROC analysis. The score showed good discrimination, with Mann–Whitney U = 5443, *p* < 0.001, and an area under the ROC curve (AUC) of 0.852 (95% CI, 0.806–0.897; *p* < 0.001). Increasing score thresholds were associated with increasing specificity for in-hospital death.

As a categorical variable, score categories were compared across vital-status groups using Fisher’s exact test, which showed a significant association between increasing score category and in-hospital death (*p* < 0.001). Additional Cox analysis was performed using 0 points as the reference category. Because the highest score categories contained few patients and produced unstable estimates, categories above 8 points may be collapsed for presentation to avoid paradoxical fluctuations in risk estimates.

## 3. Results

### 3.1. Demographic and Clinical Characteristics of Hospitalized Patients

A total of 503 patients were included in the study; 41.95% were female and 58.05% were male. Age ranged from 26 to 94 years, with a mean age of 70.54 years and a median age of 71 years (IQR 65–77). Most participants (65.81%) lived in urban areas.

Most admissions (68.79%) were through the emergency department. The median length of hospital stay (LOS) was 8 days (IQR 5–12), with a mean LOS of 9.76 days (range 1–70). Emergency admission was associated with a longer LOS (*p* = 0.019).

Clinical and demographic data were extracted from medical records. The most prevalent comorbidities were arterial hypertension (76.74%), diabetes mellitus (24.85%), atherosclerosis (21.27%), atrial fibrillation/atrial flutter (20.87%), chronic heart failure (18.49%), obesity (17.69%), and chronic coronary syndrome (13.12%), including a history of coronary angioplasty or stable angina. Valvular abnormalities were documented in 435 of 503 patients (86.48%); however, no hemodynamically significant valvular disease was recorded in the study documentation. Other comorbidities were present in 9.34% of the cohort, with stroke being the most frequent.

At discharge, 414 patients (82.31%) were discharged alive, whereas 89 patients (17.69%) died during the index hospitalization.

The demographic and clinical profile of the study cohort is summarized in Table 1.

### 3.2. Distribution of Surgical Procedures

**Figure 1:**
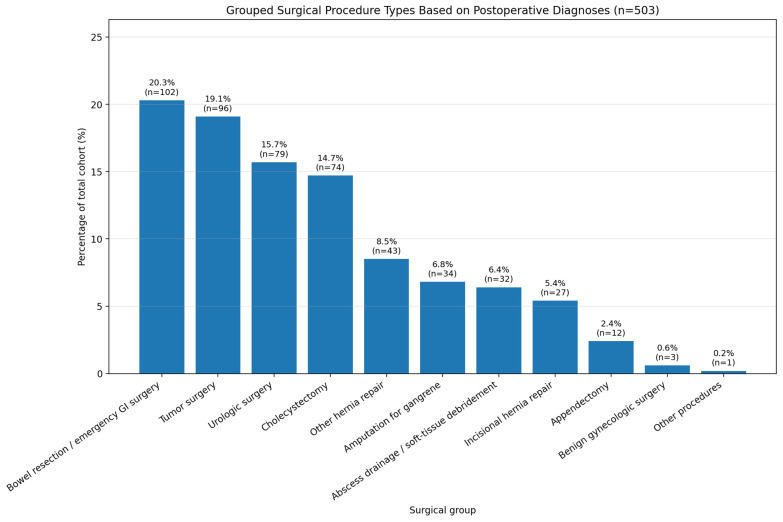
Distribution of grouped surgical procedure types based on postoperative diagnoses in the study cohort (*n* = 503); percentages are reported relative to the total cohort.

The cohort included a broad range of non-cardiac surgical procedures, reflecting real-world general surgical practice. When postoperative diagnoses were grouped according to the corresponding type of surgical intervention, the most frequent categories were bowel resection/emergency gastrointestinal surgery (20.3%), tumor surgery (19.1%), urologic surgery (15.7%), and cholecystectomy (14.7%). Smaller but clinically relevant groups included hernia repair, amputation for gangrene, abscess drainage/debridement, appendectomy, and benign gynecologic surgery (Figure 1).

### 3.3. Preoperative Echocardiographic Characteristics

The study population had a mean LVEF of 57.62% (range 25–65%), with a median of 60% (IQR 55–60%). Diastolic function parameters showed a mean peak early diastolic transmitral inflow velocity (E wave) of 104 cm/s (range 40–220 cm/s) and a median of 100 cm/s (IQR 76–121 cm/s). The mean peak late diastolic transmitral inflow velocity (A wave) was 76 cm/s (range 22–162 cm/s), with a median of 72 cm/s (IQR 56–94 cm/s). The mean E/A ratio was 1.62 (range 0.25–5.04), with a median of 1.44 (IQR 0.97–2.07).

Regarding longitudinal function, mean MAPSE was 12.05 mm (range 5.5–19.7 mm), with a median of 12.0 mm (IQR 10.8–13.2 mm). For the right ventricle, mean TAPSE was 22.05 mm (range 13.9–29.9 mm), with a median of 22.1 mm (IQR 20.1–23.9 mm). Detailed echocardiographic characteristics are presented in Table 2.

### 3.4. Perioperative Cardiovascular Risk Stratification in Non-Cardiac Surgery

#### 3.4.1. Performance of Risk Scores

The Lee/RCRI and AUB-HAS2 indices were analyzed on their original raw 0–6 scales. In ROC analysis, both comparator scores showed good discriminatory performance for in-hospital mortality. Lee/RCRI achieved an AUC of 0.860 (95% CI 0.818–0.901; *p* < 0.001), whereas AUB-HAS2 achieved an AUC of 0.779 (95% CI 0.731–0.826; *p* < 0.001). Comparator-specific optimal cut-offs with sensitivity and specificity were not emphasized because these established tools are commonly interpreted using predefined ordinal categories, and study-specific ROC thresholds would be sample-dependent.

**Figure 2:**
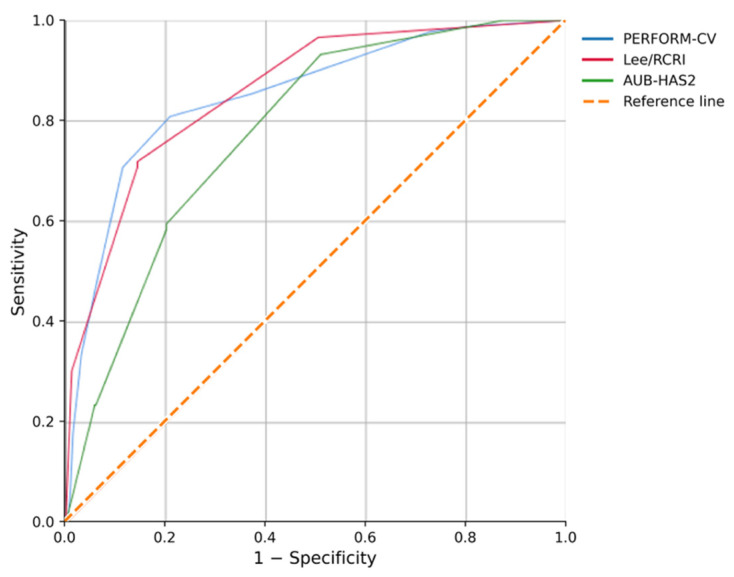
Receiver operating characteristic (ROC) curves comparing PERFORM-CV, Lee/RCRI, and AUB-HAS2 for predicting in-hospital mortality.

When assessed as an individual echocardiographic marker, MAPSE showed modest discriminatory ability for in-hospital mortality (AUC = 0.608; *p* = 0.001). The ROC-derived cut-off value of ≤10.02 mm yielded a sensitivity of 53.9% and a specificity of 61.1% (Figure 2).

#### 3.4.2. Factors Associated with In-Hospital Mortality

Univariable Cox analysis identified emergency presentation (*p* < 0.001), chronic heart failure (*p* < 0.001), atrial fibrillation (*p* < 0.001), and atherosclerosis (*p* = 0.044) as significant clinical correlates. Laboratory and imaging variables were also associated with the outcome, including higher serum creatinine (*p* < 0.001), anemia (*p* = 0.003), hyperglycemia (*p* = 0.003), lower LVEF (*p* < 0.001), and lower MAPSE (*p* < 0.001). Because both LVEF and MAPSE were associated with the endpoint, their relationship was further explored. LVEF and MAPSE showed a statistically significant but only moderate correlation (Spearman’s rho = 0.309, *p* < 0.001; Pearson r = 0.397, *p* < 0.001), and collinearity was low (VIF = 1.187).

In the multivariable model, emergency presentation (HR = 6.536, 95% CI 2.570–16.623; *p* < 0.001), chronic heart failure (HR = 6.117, 95% CI 2.993–12.501; *p* < 0.001), and increased serum creatinine (HR = 1.619, 95% CI 1.259–2.081; *p* < 0.001) remained independently associated with in-hospital mortality.

#### 3.4.3. Development and Performance of the PERFORM-CV Score

A composite risk score, PERFORM-CV (PERioperative Factors Outcomes for Risk of Mortality—CardioVascular), was developed. The final score incorporated seven variables retained on clinical and statistical grounds: emergency presentation, left ventricular ejection fraction ≤ 54%, serum creatinine ≥1.775 mg/dL, atrial fibrillation, chronic heart failure, hemoglobin ≤10.25 g/dL, and MAPSE ≤10.02 mm. Emergency presentation, renal dysfunction, and chronic heart failure were assigned 2 points each, whereas the remaining predictors were assigned 1 point each, yielding a total score ranging from 0 to 10 (Table 3). Comparative ROC results are shown in Table 4. The PERFORM-CV score showed good apparent discrimination (AUC = 0.852, *p* < 0.001) and achieved a sensitivity of 80.9% and a specificity of 79.1% at the optimal derivation-cohort cut-off of ≥3.5 points.

Specificity increased with higher PERFORM-CV values, reaching 93.5% at ≥6 points and 99.5% at 10 points. The exact ROC-derived cut-off of 3.5 points was used for score derivation and statistical reporting; for pragmatic bedside interpretation, three rounded categories were defined: low risk (0–2 points), intermediate risk (3–5 points), and high risk (≥6 points). Observed mortality increased across these categories in the derivation cohort.

As shown in Table 4, the PERFORM-CV score demonstrated good discriminatory performance (AUC = 0.852), comparable to the Lee/RCRI score. At the optimal derivation-cohort cut-off (≥3.5 points), PERFORM-CV achieved the highest sensitivity (80.9%) among the evaluated models, with a specificity of 79.1%. Specificity increased at higher PERFORM-CV thresholds, reaching 93.5% at ≥6 points and 99.5% at 10 points. For clinical interpretation, three rounded categories were defined: low risk (0–2 points), intermediate risk (3–5 points), and high risk (6–10 points). Exploratory rank-correlation analysis also showed a moderate positive association between increasing PERFORM-CV values and in-hospital death (Spearman’s rho = 0.475, *p* < 0.001), supporting the ordinal behavior of the score in the derivation cohort.

## 4. Discussion

Perioperative management of patients undergoing non-cardiac surgery remains challenging, particularly when attempting to identify individuals at increased risk of in-hospital mortality [4,5,19,20]. In this study, we evaluated the performance of established risk indices and developed PERFORM-CV, a multiparametric score integrating clinical, laboratory, and echocardiographic variables to improve risk stratification for in-hospital all-cause mortality. The rationale for this approach was that perioperative risk is not determined solely by comorbidity labels, but also by the current functional expression of cardiovascular disease, including myocardial reserve, rhythm status, renal dysfunction, anemia, and the acute physiological stress associated with admission.

In this cohort, the Lee/RCRI score also showed good discriminatory performance (AUC = 0.860), whereas AUB-HAS2 showed lower discrimination (AUC = 0.779) (Table 5). The derivation of PERFORM-CV was motivated by the need to incorporate physiological markers that may better reflect current cardiovascular status than history-based scores alone. In this regard, the present data suggest that a hybrid score combining history-based and measurement-based variables can achieve clinically useful discrimination while remaining simple enough for bedside application.

A notable aspect of the present model is the inclusion of mitral annular plane systolic excursion (MAPSE). Although MAPSE and LVEF are physiologically related and are expected to correlate in routine echocardiographic practice, they are not interchangeable. MAPSE primarily reflects longitudinal LV systolic shortening, whereas LVEF reflects global volumetric systolic performance [21,22,23]. Previous studies have shown moderate-to-strong correlations between MAPSE and LVEF while also emphasizing the complementary value of MAPSE, particularly in detecting longitudinal dysfunction that may not be fully captured by LVEF alone [13,15,21,24,25,26]. In our cohort, the association between MAPSE and LVEF was statistically significant but only moderate (Spearman’s rho = 0.309, *p* < 0.001; Pearson r = 0.397, *p* < 0.001). Moreover, the VIF was low (1.187), arguing against clinically meaningful multicollinearity. Accordingly, LVEF and MAPSE were retained in the final score as complementary rather than competing echocardiographic markers, with LVEF reflecting global systolic performance and MAPSE capturing longitudinal dysfunction that may reveal subclinical impairment not fully represented by LVEF alone.

PERFORM-CV enabled stratification into low-, intermediate-, and high-risk categories, facilitating bedside interpretation. Variables with the strongest associations in the multivariable analysis, particularly emergency presentation, chronic heart failure, and renal dysfunction, were weighted more heavily in the final score. The inclusion of objective measures such as creatinine, hemoglobin, and echocardiographic parameters may help identify patients who require closer perioperative monitoring. In our analysis, specificity increased at higher PERFORM-CV thresholds, suggesting potential utility for identifying patients at particularly high risk of in-hospital mortality (Table 6). The thresholds used to define score components were derived statistically from ROC analyses and should not be interpreted as definitive treatment or triage thresholds without external validation; however, the rounded PERFORM-CV categories (0–2, 3–5, and ≥6 points) were intended to provide a more pragmatic bedside framework. In addition, the score showed a moderate positive monotonic association with mortality status in exploratory Spearman analysis, supporting its ordinal structure beyond ROC-based discrimination alone. These findings should nevertheless be interpreted as derivation-cohort results, and the score should not be viewed as a substitute for comprehensive clinical judgment; validation in independent populations is still required.

## 5. Limitations

This study has several limitations. First, it was a two-center cohort conducted in general surgery departments. Although surgical procedures were grouped into clinically relevant categories, more granular stratification by surgical subspecialty, oncologic status, anesthetic technique, perioperative setting, and standardized preoperative severity indices was not uniformly available. This limits assessment of generalizability across specific non-cardiac surgical populations.

Second, the score was derived and evaluated in the same cohort; therefore, the reported performance reflects apparent derivation-cohort performance rather than formal internal validation, and external validation in an independent cohort remains necessary.

Third, although Cox regression was used to account for variation in the length of hospitalization, the proportional hazards assumption was not formally assessed. Accordingly, the time-to-event findings should be interpreted with caution.

Fourth, the echocardiographic assessment was designed as a focused bedside POCUS-based evaluation rather than a comprehensive advanced echocardiographic examination. Consequently, advanced echocardiographic parameters such as GLS, tissue Doppler indices, E/e′, and systematic quantitative grading of individual valvular lesions, particularly mitral regurgitation, were not consistently available and were therefore not included in the model. As a result, diastolic dysfunction and valvular disease may have been incompletely characterized.

Fifth, although valvular abnormalities were frequently documented, no hemodynamically significant valvular disease was identified in the available records, and standardized quantitative grading of these lesions was not consistently available for all patients.

Sixth, the event rate and the number of predictors included in the final model may have affected model stability and generalizability.

Seventh, formal calibration analysis was not performed in this derivation study; therefore, the present results emphasize discrimination rather than agreement between predicted and observed risk.

Eighth, all focused echocardiographic examinations were performed by a single operator. Although this reduced interobserver variability, it did not allow formal assessment of interobserver reproducibility, and the timing and availability of perioperative imaging were not fully uniform across all patients.

Finally, the ROC-derived cut-offs were data-driven and optimized for the present cohort. Although these thresholds may be useful for risk stratification within this dataset, external validation is required before they can be considered clinically applicable. In addition, although correlation and VIF analyses did not suggest meaningful collinearity between LVEF and MAPSE, a formal sensitivity analysis excluding one of the two parameters was not performed.

Further prospective studies with broader surgical phenotyping, calibration analysis, resampling-based internal validation, and external validation in independent cohorts are warranted to confirm reproducibility and clinical applicability across diverse surgical settings. In addition, because a substantial proportion of the cohort underwent oncologic procedures, perioperative outcomes in these subgroups may also have been influenced by broader patient-centered and disease-specific factors that were outside the scope of the present model, including psychosocial burden [27] and complications related to systemic therapies or paraneoplastic syndromes [28].

## 6. Conclusions

**Figure 3:**
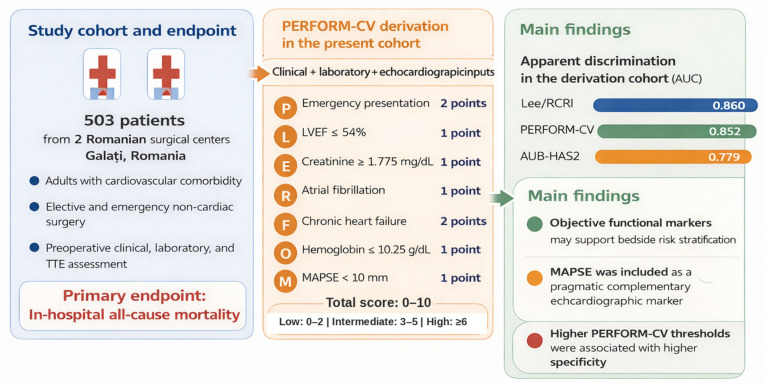
Graphical abstract of PERFORM-CV score.

The present study introduces and provides a preliminary evaluation of PERFORM-CV, a multidimensional risk-stratification tool for predicting in-hospital mortality after non-cardiac surgery that integrates clinical presentation, laboratory markers, and echocardiographic parameters, including MAPSE. In this cohort, PERFORM-CV showed good apparent discriminatory performance (AUC = 0.852, 95% CI 0.806–0.897) and may complement established tools such as Lee/RCRI by incorporating objective imaging- and biomarker-derived variables (Figure 3). The reported score thresholds should be interpreted as data-driven derivation cut-offs, whereas the rounded score categories were intended for pragmatic bedside interpretation. Further resampling-based internal validation and external validation in independent cohorts are needed before broader clinical implementation.

## Figures and Tables

**Table 1:** The demographic and clinical profile of the study cohort.

Characteristic	Category	Frequency (*n*)	Percentage (%)
Sex	Female	211	41.95
	Male	292	58.05
Residence	Urban	331	65.81
	Rural	172	34.19
Admission Type	Emergency	346	68.79
	Scheduled	157	31.21
Comorbidities			
	Arterial Hypertension	386	76.74
	Diabetes Mellitus	125	24.85
	Atherosclerosis	107	21.27
	Atrial Fibrillation/Atrial Flutter	105	20.87
	Chronic Heart Failure	93	18.49
	Obesity	89	17.69
	Chronic Coronary Syndrome	66	13.12

**Table 2:** Echocardiographic characteristics.

Parameter	Mean	Median (IQR)	Range (Min–Max)
Systolic Function			
LVEF (%)	57.62	60 (55–60)	25–65
MAPSE (mm)	12.05	12.0 (10.8–13.2)	5.5–19.7
TAPSE (mm)	22.05	22.10 (20.1–23.9)	13.9–29.9
Diastolic Function			
E wave (cm/s)	104	100 (76–121)	40–220
A wave (cm/s)	76	72 (56–94)	22–162
E/A Ratio	1.62	1.44 (0.97–2.07)	0.25–5.04

Note: LVEF—Left Ventricular Ejection Fraction; MAPSE—Mitral Annular Plane Systolic Excursion; TAPSE—Tricuspid Annular Plane Systolic Excursion.

**Table 3:** PERioperative Factors Outcomes for Risk of Mortality—CardioVascular.

Letter	Clinical Criterion	Definition	Points
P	Presentation	Emergency presentation	2
E	Ejection Fraction	LVEF ≤ 54%	1
R	Renal Function	Serum creatinine ≥ 1.775 mg/dL	2
F	Fibrillation	History of atrial fibrillation	1
O	Other Cardiac History	Chronic heart failure	2
R	Reduced Hemoglobin	Hemoglobin ≤ 10.25 g/dL	1
M	MAPSE	MAPSE ≤ 10.02 mm	1
	Total Score		0–10

Note: LVEF assessed by TTE; creatinine and hemoglobin obtained from admission laboratory testing; MAPSE measured by M-mode at the septal mitral annulus.

**Table 4:** Comparative ROC Analysis of Risk Scores for Predicting In-Hospital Mortality.

	AUC	Standard Error	*p*-Value	95% Confidence Interval		Optimal Cut-Off	Sensitivity	Specificity
PERFORM-CV	0.852	0.023	<0.001	0.806	0.897	≥3.5	80.9%	79.1%
Lee/RCRI	0.860	0.021	<0.001	0.818	0.901	≥8	71.9%	85.7%
AUB-HAS2	0.779	0.024	<0.001	0.731	0.826	≥5.65	59.6%	79.9%

Note: AUC—Area Under the Curve; CI—Confidence Interval; Std. Error—Standard Error. Comparator-specific optimal cut-offs are not shown because the primary comparison focused on overall discrimination of established categorical scores rather than on study-specific dichotomization.

**Table 5:** Comparison with existing risk scores.

Characteristic	RCRI (Lee Score)	AUB-HAS2 Score	PERFORM-CV Score
Year of development	1999	2019	Proposed in this study
Type of variables	Clinical + laboratory	Clinical + laboratory	Clinical + laboratory + echocardiographic
Echo parameters	No	No	Yes (EF, MAPSE)
Biomarkers	Creatinine	Hemoglobin (anemia)	Creatinine, hemoglobin
Cardiac variables	IHD; HF	Heart disease; angina/dyspnea	HF, AF, LV dysfunction
Renal function	Creatinine > 2 mg/dL	No	Creatinine ≥ 1.775 mg/dL
Cardiac function	No	No	Yes
Emergency surgery	No	Yes	Yes
Maximum score	6 points	6 points	10 points

**Table 6:** Variables included in PERFORM-CV and their statistical association with in-hospital mortality.

L	Risk Factor	Statistical Impact	Role in PERFORM-CV
P	Emergency Presentation	HR = 6.536; 95% CI 2.570–16.623; *p* < 0.001	Independent multivariable predictor; assigned 2 points
E	LVEF ≤ 54%	HR = 5.107; 95% CI 2.977–8.763; *p* < 0.001	ROC-derived threshold retained in the final score; assigned 1 point
R	Renal function (creatinine ≥ 1.775 mg/dL)	HR = 2.725; 95% CI 2.093–3.548; *p* < 0.001	ROC-derived threshold retained in the final score; assigned 2 points
F	Atrial Fibrillation	HR = 2.456; 95% CI 1.484–4.062; *p* < 0.001	Univariable risk factor retained in the final score; assigned 1 point
O	Other cardiac history (chronic heart failure)	HR = 6.117; 95% CI 2.993–12.501; *p* < 0.001	Independent multivariable predictor; assigned 2 points
R	Reduced Hemoglobin ≤ 10.25 g/dL	HR = 3.755; 95% CI 2.146–6.568; *p* < 0.001	ROC-derived threshold retained in the final score; assigned 1 point
M	MAPSE ≤ 10.02 mm	HR = 3.968; 95% CI 2.259–6.791; *p* < 0.001	ROC-derived threshold retained in the final score; assigned 1 point

## Data Availability

The original contributions presented in this study are included in the article. Further inquiries can be directed to the corresponding authors.

## Abbreviations

The following abbreviations are used in this manuscript:

AF	Atrial Fibrillation
ASE	American Society of Echocardiography
AUB-HAS2	American University of Beirut-Hassouna Risk Index
AUC	Area Under the Curve
BMI	Body Mass Index
CI	Confidence Interval
DT/EDT	Deceleration Time/E-wave Deceleration Time
E/A	Ratio of Peak Early to Late Diastolic Transmitral Filling Velocity
EACVI	European Association of Cardiovascular Imaging
HF	Heart Failure
HR	Hazard Ratio
IQR	Interquartile Range
LOS	Length of Hospital Stay
LVEF	Left Ventricular Ejection Fraction
MACE	Major Adverse Cardiovascular Events
MAPSE	Mitral Annular Plane Systolic Excursion
PERFORM-CV	PERioperative Factors Outcomes for Risk of Mortality—CardioVascular
POCUS	Point-of-Care Ultrasound
RCRI	Revised Cardiac Risk Index (Lee Score)
ROC	Receiver Operating Characteristic
TAPSE	Tricuspid Annular Plane Systolic Excursion
TTE	Transthoracic Echocardiography
